# Complete genome sequence of *Photobacterium damselae* strain HNDF01 isolated from diseased *Plectropomus leopardus* in Hainan, China

**DOI:** 10.1128/mra.00938-26

**Published:** 2026-09-29

**Authors:** Chen Dai, Qingming Liu, Dapeng Luo, Jing Cui, Zhigang Tu

**Affiliations:** 1 Hainan Provincial Aquatic Animal Epidemic Surveillance Center, Hainan Academy of Ocean and Fisheries Sciences 594260 , Haikou, China; 2 Hainan Provincial Key Laboratory of Tropical Maricultural Technologies, Hainan Academy of Ocean and Fisheries Sciences 594260 , Haikou, China; Montana State University, Bozeman, Montana, USA

**Keywords:** *Photobacterium damselae*, complete genome, strain HNDF01, whole-genome sequencing, *Plectropomus leopardus*

## Abstract

We report the complete genome sequence of *Photobacterium damselae* subsp. *damselae* strain HNDF01, isolated from diseased *Plectropomus leopardus* in Hainan, China. The genome consists of two circular chromosomes (4,174,829 bp) and one plasmid (160,669 bp), encoding 3,753 protein-coding genes, 217 tRNAs, 47 rRNAs, and 10 sRNAs.

## ANNOUNCEMENT

*Photobacterium damselae* is a gram-negative, facultative anaerobic marine bacterium and a major aquaculture pathogen causing hemorrhagic septicemia, skin ulcers, and high mortality in fish (1–3). It was reported in sea bass (*Dicentrarchus labrax*) (4), sea bream (*Sparus aurata*) (5), kuruma shrimp (*Penaeus japonicus*) (6), spotted seal (*Phoca largha*) (7), sea cucumber (*Apostichopus japonicus*) (8), guitarfish (*Rhinobatos rhinobatos*) (9), and striped dolphins (*Stenella coeruleoalba*) (10). Strain HNDF01 was isolated from the diseased *Plectropomus leopardus* (body/tail rot, >90% mortality within 2–3 days) at a farm in Dongfang, China (18.82°N, 108.67°E) on 2216E agar at 28°C. The pure colony was stored at −80°C. Genomic DNA was extracted from a single colony grown in 2216E medium at 28°C, 180 rpm for 16 h using a modified phenol-chloroform method. Then, genomic DNA was randomly sheared using a Covaris E220 focused-ultrasonicator (Covaris, Woburn, MA, USA). Size selection of the fragmented genomic DNA (200–400 bp) was performed using the Agencourt AMPure XP-Medium Kit (Beckman Coulter, Brea, CA, USA). Purified fragments were subjected to end repair, 3′ adenylation, and adaptor ligation. PCR products were purified, heat-denatured, and circularized via splint oligo into single-strand circle DNA, which was sequenced on DNBSEQ (formerly BGISEQ-500, BGI-Shenzhen). Size-fractionated genomic DNA was processed to generate SMRTbell template libraries via end repair and hairpin adaptor ligation and subsequently sequenced on the PacBio RSII platform. Raw DNBSEQ short-read data were filtered to remove reads with >40% low-quality (Phred ≤20) or ambiguous-N bases, adapters, and duplicates (10%–20% for small-insert and more for large-insert libraries), retaining 8,738,130 clean reads (1,167 Mb). PacBio RSII subreads from single-template ZMW reads were adapter-trimmed and filtered to ≥1,000 bp, yielding 195,292 clean subreads (mean read length, 8,546 bp; N50, 8,768 bp; and N90, 6,038 bp) (11–13). PacBio subreads were extracted from raw polymerase reads using SMRT Analysis v2.3.0 (Pacific Biosciences). Error-corrected reads were independently assembled with Canu v1.5 and Falcon v0.3.0 (14, 15). Assemblies were evaluated separately without merging, and the best draft was selected by continuity, gap status, and circular replicon evidence. Single-base error polishing of the draft assembly was performed using DNBSEQ short-read data with GATK v3.4 (16). All replicons were assembled into a single, gapless, N-free contig, with chromosomes and the plasmid discriminated by sequence length, GC content, and marker genes. DNBSEQ short-read polishing and uniform read-depth coverage further confirmed the closed, complete genome assembly. The total size of the *P. damsalae* HNDF01 genome is 4.33 Mb, including two chromosomes of 2,950,689 bp (41.28% GC) and 1,224,140 bp (38.99% GC), as well as a single plasmid of 160,669 bp (43.44% GC). Gene prediction was applied using Glimmer 3.02 (17). The genome contained 3,753 protein-coding genes, alongside a set of non-coding RNAs including 217 tRNAs, 17 5S rRNAs, 15 16S rRNAs, 15 23S rRNAs, and 10 sRNAs. Average nucleotide identity analysis (fastANI v1.32) (18) was performed for taxonomic identification to determine the species-level relationship between the target strain and reference genomes. Comparison with *P. damselae* subsp. *damselae* CSP DAM1 (GCA_021766015.1) and *P. damselae* subsp. *damselae* KC-Na-NB1 (GCA_004135105.1) strains revealed 98.29% and 97.22%, respectively. Together, these results confirm that strain HNDF01 is *Photobacterium damselae*.

## Data Availability

All data are available at GenBank under BioProject accession number PRJNA1484815. The BioSample accession number is SAMN61258053. The raw BGI and PacBio sequencing data can be found accession under number SRR39720355 and SRR39720354, respectively. The genome assembly has been deposited in GenBank under the whole-genome shotgun (WGS) accession JCASQK000000000.
